# Editorial: Polymer Chemistry Editor’s Pick 2021

**DOI:** 10.3389/fchem.2021.718755

**Published:** 2021-06-30

**Authors:** Pellegrino Musto

**Affiliations:** Institute on Polymers, Composites and Biomaterials, National Research Council (CNR), Pozzuoli, Italy

**Keywords:** hydrogel, membrane, surface properties, polymer scaffold, light-responsive materials, biosensors

**Editorial on the Research Topic**

**Polymer Chemistry Editor’s Pick 2021**

The section *Polymer Chemistry* of the journal *Frontiers in Chemistry* was born with the scope of witnessing, disseminating and fostering the advancements of the discipline of macromolecular science in its broadest significance. The opening of a specific section devoted entirely to this subject was justified by the profound impact this discipline and the outcomes of its research efforts have had and continue to have on our everyday life. As stated in the inaugural article (Musto) “Despite the astonishing achievements we have witnessed along the years, many exciting challenges remain to be faced, including green polymer chemistry, environmental pollution issues, polymers for energy storage and delivery, polymers for the human health.” Seven years after its launch, we may say that, so far, the section has accomplished the tasks it was created for. The readership is growing, as well as the community of editors and qualified authors. Many interesting papers have appeared, addressing the most relevant challenges currently open, with special emphasis on health related issues. It was therefore decided, according with the editorial office, that the time was mature to realize a Research Topic, assembling a collection of articles that could give to the reader an outlook of the section activity and a summary of its main achievements. The choice among the numerous high-quality contributions was not easy; it was driven by the limited number of articles to be included (thirteen), the readership acceptance as evaluated by the significant bibliometric figures made available by the journal platform and, of course, the personal taste of the Editor, of which he takes full responsibility. The work presented herein witnesses the broad range of activities covered by the section and demonstrates strong advances in theory, experiment and methodology applied to forefront research challenges.

The first contribution Si et al. deals with the development of an innovative hydrogel to be used for selective removal of aromatic pollutants from wastewater. This stimuli-responsive, molecularly imprinted material was characterized by spectroscopic and electron microscopy means and was investigated in terms of adsorption and selective recognition of substituted phenols. It was demonstrated that the hydrogel has good selectivity, temperature switching properties, and is reusable, which makes it a good candidate for controlled separation and release of phenolic pollutants.

In the second paper Ding et al. is described the synthesis of chitosan grafted by β-cyclodextrin. This functional material exhibited strong antimicrobial activity against *E. coli* and *Staphylococcus xylosus*, which was tuned by adjusting the amino content of the polysaccharide. The reported results may be relevant in the livestock industry as a means of reducing the dosage of antibiotics and the antibiotic residues in animal-derived foods.

Next, Galizia and Bye present a detailed review on organic solvent nanofiltration, highlighting the relationship among this process and the underlying physical-chemistry and polymer chemistry. In the first part of the review are discussed the available theoretical models, along with some misleading conclusions commonly encountered in the literature. The following section describes the most conventional materials currently in use and identifies a number of alternative materials that may impact this technology in the near future.

The work by Pizarro et al. is an excellent example of the application of precision synthetic routes, in particular, atom-transfer radical polymerization (ATRP), to finely control the surface properties of polymer films. It was demonstrated that parameters such as pore size, roughness, thickness, and wettability of a co-polymer film could be varied by changing the co-monomer structures. Moreover, thermal annealing was found to improve significantly the surface quality, thus providing a further means toward surface engineering.

The review article by Yao et al. is an account of current research on polymer-based composites to be used as electrolytes in Lithium batteries. This is a relevant technological challenge, since substitution of the conventional liquid electrolytes currently in use with solid-state components may allow to overcome numerous weaknesses of Li-ion cells. The survey describes in detail the main classes of composites under consideration, with the relative conductivity mechanisms. The fundamental issues still unsolved are critically discussed.

The research paper by Quigley et al. concerns a relevant health issue, namely the repairing of Volumetric Muscle Loss (VML) as a consequence of trauma (road/industrial accident, war injury) or disease (muscular dystrophy, muscle atrophy). The authors report an innovative biosynthetic material based on the “Trojan Horse” concept. It is an alginate/myoblast construct that was tested successfully as a scaffold for remodelling of diseased and/or damaged muscle.

The work by Talebian et al. deals with electrically conductive hydrogels to be used as biofibers for *in-vivo* stimulation of electrically excitable cells. The biofibers were realized by electrospinning of an alginate/graphene nanocomposite. The graphene-additivated biofibers exhibited better mechanical, electrical and electrochemical properties in comparison to the pristine fibers. In the light of the results obtained, they were also proposed as 3D scaffolds for tissue engineering applications.

Light-responsive materials, e.g., materials that exhibit on-off switching properties when irradiated at specific frequencies, are a hot-topic for an increasing number of high-tech applications. The paper by Pang et al. reports on the realization and the morphological/spectroscopic characterization of one of such systems based on the interaction between β-cyclodextrin and the azobenzene group. This system was shown to self-assemble under the specific stimulus, forming nanoparticles whose dimensions can be carefully controlled by polymer composition and irradiation. Interesting applications were demonstrated as nanocarrier for the precise delivery of anticancer drugs.

Zheng et al. report the preparation and remarkable performances of a proton-exchange membrane made by a composite material. The filler was a MOF (Metal Organic Framework), a recently developed class of molecular structures that is gaining a prominent role in contemporary materials science. The cage structure of the MOF was covalently cross-linked to the polymer matrix, imparting to the composite-membrane excellent thermal and dimensional stability. The filled membrane showed a proton conductivity considerably improved with respect to the pristine matrix.

Lin et al. present a comprehensive computational study aimed at simulating the kinetics of the photopolymerization process. Taking into account both radical-mediated and oxygen-mediated reaction pathways, the authors were able to account for the relevant process parameters (e.g., photosensitizer concentration, oxygen concentration, light dose and intensity). They derived analytical equations to evaluate curing efficacy and curing depth. These formulas were successfully tested on microfabricated reactive systems.

The review article by Wenrui et al. is an account of the carbon-based composites used in the specialized field of flexible biosensors. The main routes to Carbon Fiber functionalization are reviewed, with special emphasis on those involving noble metals and metal oxides, polymers and MOFs. A final section describes two key-applications of these fibers, e.g., as electrochemical biosensors and as flexible or wearable biosensors.

Liang et al. describe the preparation of a bone repair scaffold made by poly(glycerol-sebacate), a synthetic polymer exhibiting good biocompatibility and high elasticity. Optimizing the photocrosslinking process and the preparation protocol, a sponge-like structure was realized, which improved hydrophilicity and promoted vascularization and osteogenesis. The potential of the proposed material to serve as a bone-mimicking scaffold was demonstrated.

Belda Marín et al. reviewed silk polymer nanocomposites and their applications as biomaterials. Silk fibroin is a versatile material that has found increasing applications for its biocompatibility, biodegradability and excellent mechanical properties. Furthermore, it can be readily processed in various forms at the macro-, micro and nano-scales through additive manufacturing techniques such as 3D-printing bioprinting or stereolithography. Filling the silk fibroin with inorganic nanoparticles provides further versatility and novel functional properties such as antibacterial activity, fluorescence properties, UV protection. The two main routes to bionanocomposite formulation are reviewed, e.g., *in situ* synthesis of INPs in silk materials, and the addition of preformed INPs to silk materials, along with their strengths and weaknesses. In the final section, an overview is provided of the present and perspective applications of these biomaterials.

